# Utilization of care and alignment of screening with NCCN guidelines for patients with Lynch syndrome: a retrospective cohort study

**DOI:** 10.1186/s13053-026-00334-1

**Published:** 2026-03-19

**Authors:** Christina Lowry, Cecile Laurent, Ameek K. Bindra, C. Bethan Powell, Christine Garcia

**Affiliations:** 1 Kaiser Permanente San Francisco, Obstetric and Gynecologic Residency Program, 2836 Latham Drive, Sacramento, San Francisco, CA 95864 USA; 2 Kaiser Permanente, Division of Research Northern California, Pleasanton, CA USA; 3 Carle Illinois College of Medicine, University of Illinois Urbana-Champaign, Urbana, IL USA; 4 Division of Gynecologic Oncology, University of California San Francisco, San Francisco, CA USA; 5 Kaiser Permanente, Division of Gynecologic Oncology, San Francisco, CA USA

**Keywords:** Lynch syndrome, Cancer screening, National Comprehensive Cancer Network (NCCN) guidelines, Genetic testing, Hereditary cancer screening, Pathogenic variants

## Abstract

**Objectives:**

Determine the proportion of patients with Lynch syndrome who received guideline-concordant care according to NCCN guidelines during the study period (2015–2018).

**Methods:**

A retrospective cohort study was performed at Kaiser Permanente Northern California including patients with germline pathogenic variants in MLH1, MSH2/EPCAM, MSH6 or PMS2 tested between 01/01/2015 and 12/31/2018. Clinical and demographic characteristics were abstracted from chart review. Screenings were assessed for gynecologic, gastrointestinal (GI), urinary, skin, and neurologic cancer sites. Concordance was assessed against NCCN guidelines (versions 1.2015 through 1.2018) matched to the year of each patient encounter. The primary outcome was receipt of guideline-concordant care. Secondary outcomes included rates of under- and over-utilization and clinical/demographic factors associated with care utilization.

**Results:**

We identified 234 patients: 46 with pathogenic MLH1 variants, 64 with MSH6, 83 with PMS2 and 41 with MSH2/EPCAM variants. Median age was 52 years (IQR: 39–62), most were white (60%) and 29% were tested based on a personal cancer history. Half (48%) of patients received screening as recommended by NCCN guidelines, with highest concordance for GI cancer screening. A quarter of patients received less screening than recommended by guidelines. Patients with MLH1 variants had higher rates of transvaginal ultrasound (34.2%) and urologic cancer screening (28.3%) compared to those with other variants.

**Conclusions:**

Only 48% of patients in this integrated healthcare system received guideline-concordant Lynch syndrome surveillance. Our findings highlight variation in screening practices across pathogenic variant types and suggest opportunities for improving adherence to recommended screenings within healthcare systems.

## Background

Lynch syndrome, also known as hereditary nonpolyposis colorectal cancer, is one of the most common hereditary cancer syndromes, with prevalence estimates as high as 1 in 279 individuals [1, 2]. This condition is inherited in an autosomal dominant manner and caused by germline pathogenic variants in four mismatch repair (MMR) genes: MLH1, MSH2, MSH6, and PMS2 [1].

Carriers of pathogenic variants in these genes are at high lifetime risk for colorectal and endometrial cancers as well as increased risks for other extracolonic tumors. The continuum of this disorder includes tumors of the ovaries, small bowel, urothelium, pancreas, biliary tract, and stomach [3]. Cancer risks vary substantially by gene: women with pathogenic MLH1 or MSH2 variants have 40–50% lifetime risk of colorectal cancer, 40–60% risk of endometrial cancer and 10–12% risk of ovarian cancer, while those with MSH6 or PMS2 variants have lower penetrance [4, 5].

The National Comprehensive Cancer Network (NCCN) provides guidelines for management and risk reduction strategies for Lynch syndrome. A challenge has been frequent updates in guidelines, unclear benefits of surveillance strategies for rare cancer types, and individualizing cancer risk due to individual patient factors and variant type. Since 2016, NCCN recommendations have been distinguished by specific pathogenic variant [6].

Some guidelines have strong evidence supporting earlier detection and improved outcomes. For example, colonoscopies every 1–2 years starting as young as 20 years old for MLH1 carriers have been shown to have an all-cause mortality benefit with earlier detection of colorectal cancers [7]. Similarly, risk-reducing surgery such as total hysterectomy and bilateral salpingo-oophorectomy has been shown to decrease the lifetime risk of endometrial and ovarian cancer, however timing of this surgery continues to be debated and varies by variant [4]. Recent data from the Prospective Lynch Syndrome Database (PLSD) suggest that risk-reducing gynecologic surgery before age 40 provides minimal mortality benefit, and premenopausal oophorectomy in MSH6 and PMS2 carriers has no measurable mortality benefit [8]. In contrast, no recommendation has been made for routine detection of urothelial cancers, though NCCN states annual urinalysis can be considered [5].

Due to the complexities of screening for patients with Lynch syndrome, we sought to determine the level of appropriate screening in a large integrated health care system. The primary objective of our study was to identify the proportion of patients with Lynch syndrome receiving NCCN guideline-concordant care. Our secondary objectives were to assess over- and under-utilization of screening tests and to determine factors associated with screening practices. Lastly, we determined the rate of screening tests detecting new malignancies. We hypothesized that given the complexity of recommendations and potential screenings, most patients would not be receiving adequate screening for their variant-specific cancer risks.

## Methods

This study was conducted at Kaiser Permanente Northern California (KPNC), which includes over 4 million members with demographics mirroring the larger geographic population [9]. Kaiser Northern California is a not-for-profit health management organization which provides integrated healthcare to patients and has a long-standing electronic medical record (EMR). The cohort was identified from a genetic database of patients who were over age 18 years and tested positive for a pathogenic or likely pathogenic variant in MLH1, MSH2/EPCAM, MSH6 or PMS2 from January 2015 through December 2018. Screening and management for patients with Lynch syndrome is divided amongst providers in our system, with gastroenterologists managing screening for gastrointestinal cancers (GI) with esophagogastroduodenoscopy (EGD) and colonoscopy, gynecologists managing uterine and ovarian risks, and primary care doctors responsible for management and referral for other cancers.

Clinical and demographic characteristics were abstracted from the EMR and chart review. We collected personal, diagnostic, and clinical characteristics including age at diagnosis, race, ethnicity, personal cancer history, cancer site, family history of cancer, cancer stage, comorbidities, gravidity, and parity.

For the management of gastrointestinal cancer risks we collected data on the following screening modalities: helicobacter pylori (H. pylori) tests, EGD, colonoscopy, magnetic resonance cholangiopancreatography (MRCP) and endoscopic ultrasound (EUS). For gynecologic cancer risks we collected rates of transvaginal ultrasound (TVUS), Cancer Antigen-125 (CA-125) from blood draws, endometrial biopsies (EMB), hysterectomy, and bilateral salpingo-oophorectomies (BSO). For urothelial cancer risk, we collected data on urinalysis/urology referral, renal ultrasound, and urine cytology/cystoscopy. Additional screening tests collected were skin exam/dermatology referral, brain magnetic resonance imaging (MRI) and neurology exam/neurology referral.

Guideline concordance definition: We defined utilization of care based on NCCN guidelines. For each patient encounter, concordance was assessed against the NCCN guideline version corresponding to that calendar year (versions 1.2015, 1.2016, 2.2016, 1.2017, 3.2017, and 1.2018). Key recommendations included: colonoscopy every 1–2 years; consideration of EGD with extended duodenoscopy every 3–4 years; consideration of annual urinalysis in MSH2 carriers; and consideration of endometrial surveillance (TVUS, EMB) or risk-reducing hysterectomy/BSO in women who have completed childbearing.

If patients completed a screening test which was “recommended” or “could be considered” per NCCN guidelines for a given year at the recommended frequency, patients were considered to have guideline-concordant care (“same utilization” in our analysis). If patients did not receive screening exams or received screenings at intervals less frequently than recommended, this was characterized as under-utilization. For example, if a patient with a pathogenic MLH1 variant had a colonoscopy procedure but less often than every 2 years this was considered under-utilization. If patients had screening tests beyond what “could be considered” per NCCN guidelines or at an increased frequency, that was classified as over-utilization. Tests performed for symptoms or follow-up of abnormal results were not included as a screening test.

Statistical methods: Statistical analyses were conducted using SAS version 9.4 (SAS Institute Inc., Cary, NC, USA). We compared various demographic characteristics for cancer patients by Lynch syndrome gene and by specific screening modalities according to the cancer risk. For categorical variables, we calculated frequencies and percentage distributions. For continuous variables, we calculated medians and interquartile ranges. We used Fisher’s exact test to calculate p-values for categorical variables with small cell counts, and Pearson’s chi-square test with larger cell counts. We used the Kruskal-Wallis test to calculate p-values for continuous variables.

This study received approval from the KPNC Institutional Review Board.

## Results

Of the 234 patients with Lynch Syndrome, the most common pathogenic variant was PMS2 (35%, 83/234) followed by MSH6 (27%, 64/234), MLH1 (20%, 46/234) and MSH2/EPCAM (18%, 41/234). The median age at time of genetic testing was 52 years old and a majority of the study population identified as female (94%, 219/234) and of white race/ethnicity (60%, 140/234). The median age at genetic testing differed significantly by variant type, with MLH1 carriers being younger and MSH6 carriers being older than other variant carriers. 29% of patients had testing due to a personal history of cancer. This was more likely in those with MLH1 and MSH2/EPCAM variants compared to MSH6 and PMS2 variants (Table 1).

**Table 1:** Lynch Syndrome Cohort Demographics

	MLH1	MSH2/EPCAM	MSH6	PMS2	Total	P-values
**N**	46	41	64	83	234	
**Median age at test (yrs) (IQR)**	46 (33-54)	52 (42-63)	57 (45-64)	53 (39-65)	52 (39-62)	0.003
**Sex**						0.1
Male	5 (10.87)	4 (9.76)	5 (7.81)	1 (1.2)	15	
Female	41 (89.13)	37 (90.24)	59 (92.19)	82 (98.8)	219	
**Race/ethnicity**						0.17
Asian	9 (19.57)	12 (29.27)	10 (15.63)	9 (10.84)	40	
Black	3 (6.52)	2 (4.88)	1 (1.56)	1 (1.2)	7	
Hispanic	5 (10.87)	5 (12.2)	4 (6.25)	7 (8.43)	21	
Other/unknown	6 (13.04)	5 (12.2)	8 (12.5)	7 (8.43)	26	
White	23 (50)	17 (41.46)	41 (64.06)	59 (71.08)	140	
**Indication for test**						**< 0.01**
Personal cancer	15 (32.61)	14 (34.15)	15 (23.44)	24 (28.92)	68	
Family cancer	14 (30.43)	7 (17.07)	17 (26.56)	40 (48.19)	78	
Diagnostic/Family History	15 (32.61)	20 (48.78)	32 (50)	19 (22.89)	86	
Other/Unknown	2 (4.35)	0 (0)	0 (0)	0 (0)	2	
Cancer dx*/**						
Colon	14 (30.43)	12 (29.27)	14 (21.88)	16 (19.28)	56	0.42
Uterine	7 (15.22)	16 (39.02)	17 (26.56)	9 (10.84)	49	**0.02**
Other	4 (8.7)	5 (12.2)	13 (20.31)	15 (18.07)	37	0.33
None	23 (50.0)	12 (29.27)	23 (35.94)	45 (54.22)	103	**0.02**
**Year of genetic test**						0.81
2015	9 (19.57)	6 (14.63)	6 (9.38)	13 (15.66)	34	
2016	11 (23.91)	8 (19.51)	16 (25)	22 (26.51)	57	
2017	13 (28.26)	12 (29.27)	20 (31.25)	17 (20.48)	62	
2018	13 (28.26)	15 (36.59)	22 (34.38)	31 (37.35)	81	
**Parity (Limited to women)**						0.74
Parous	29 (70.73)	25 (67.57)	43 (72.88)	63 (76.83)	161	
Non-parous	12 (29.27)	12 (32.43)	16 (27.12)	19 (23.17)	59	

* diagnosis prior to genetic test

** 12 patients have multiple cancers

For our primary outcome, we found that 48% (113/234) of patients received screening or surveillance in concordance with NCCN guidelines. A quarter of patients (58/234) received more screening or surveillance than recommended and 20% (45/234) received less than recommended. The remaining study participants received excessive screening in some areas and insufficient screening in others.

Compliance for gynecologic cancers screening includes TVUS, endometrial biopsies (EMB) at variant-specific intervals or hysterectomy/BSO after childbearing. We found that 22% (48/219) of female patients received TVUS, EMB, or hysterectomy and/or BSO screening tests in accordance with NCCN guidelines (Table 2). 28% of female patients underwent hysterectomy and/or BSO during the study period which would also be considered guideline-concordant care.

**Table 2:** Gynecologic Cancer Risk Factors and Rates of Gynecologic Cancer Screening

	TVUS*	p-value	Ca 125*	p-value	EMB^	p-value	Hysterectomy^	p-value	BSO*	p-value
**N**	45		46		49		54		57	
**Median age**	48 (42-55)		50 (41-56)		47 (40-55)		50 (42-55)		51 (44-56)	
Gene mutation**		0.12		0.19		0.36		0.68		0.88
MLH1	14 (34.15)	0.02	13 (31.71)	0.06	11 (26.83)	0.45	9 (21.95)	0.66	9 (21.95)	0.51
MSH2/EPCAM	6 (16.22)	0.47	5 (13.51)	0.22	6 (16.22)	0.32	8 (21.62)	0.64	9 (24.32)	0.8
MSH6	10 (16.95)	0.42	10 (16.95)	0.37	10 (16.95)	0.24	18 (30.51)	0.22	17 (28.81)	0.57
PMS2	15 (18.29)	0.52	18 (21.95)	0.79	22 (26.83)	0.22	19 (23.17)	0.69	22 (26.83)	0.83
**Race/ethnicity**		0.09		0.53		0.23		0.41		0.36
Asian	3 (6.67)		4 (8.7)		5 (10.2)		8 (14.81)		7 (12.28)	
Black	3 (6.67)		2 (4.35)		0 (0)		0 (0)		0 (0)	
Hispanic	7 (15.56)		5 (10.87)		7 (14.29)		7 (12.96)		7 (12.28)	
Other/unknown	4 (8.89)		4 (8.7)		5 (10.2)		7 (12.96)		6 (10.53)	
White	28 (62.22)		31 (67.39)		32 (65.31)		32 (59.26)		37 (64.91)	
**Family history ovarian cancer**		0.82		0.61		0.53		0.6		0.69
Yes	3 (6.67)		2 (4.35)		2 (4.08)		4 (7.41)		4 (7.02)	
No	42 (93.33)		44 (95.65)		47 (95.92)		50 (92.59)		53 (92.98)	
**Family history uterine cancer**		0.33		0.31		0.82		0.39		0.84
Yes	4 (8.89)		4 (8.7)		6 (12.24)		9 (16.67)		8 (14.04)	
No	41 (91.11)		42 (91.3)		43 (87.76)		45 (83.33)		49 (85.96)	

* Includes patient with at least one ovary and over age 30yrs

^ Includes patients with intact uterus and over age 30yrs

** Row percentage calculated

When evaluating the screening utilization for GI cancers, 40% of patients (93/234) had screening as recommended by NCCN. Colonoscopy was the most common screening tool with 59% of patients being screened (139/234). The second most common test performed was an EGD (49%, 114/234), followed by H. pylori testing (22%, 51/234). Patients with a pathogenic MLH1 variant were significantly more likely to have screening performed with an EGD.

Overall, 18 patients received a new cancer diagnosis over the study period and 16 were detected by abnormal screening tests which were performed as part of recommended surveillance.

## Discussion

Our study found that only 48% of patients in an integrated healthcare system received guideline-concordant Lynch syndrome surveillance. Guideline-concordant care was most consistent for common cancers, such as uterine and colon cancer, and in patients with MLH1 variants, who generally have higher lifetime cancer risks and the earliest age of onset. These cancers benefit from years of research and well-established risk reduction strategies, particularly for patients with the highest-risk variants, MLH1 and MSH2. The clarity and robustness of these guidelines likely contribute to better adherence among healthcare providers and patients.

Multiple factors may contribute to the observed under-utilization and over-utilization of care. These factors are present at the patient, provider, and healthcare system levels. Patient-level barriers include the emotional and physical burden of intensive surveillance protocols, practical demands such as time off work and family obligations, and varying risk perception [10]. Provider-level barriers include limited familiarity with Lynch syndrome surveillance recommendations, particularly among primary care providers who may have only one Lynch syndrome patient per 1,800-patient panel, and inconsistent recommendations across specialists [10]. Healthcare system barriers may include billing complexities and lack of coordinated care systems with reminder mechanisms [10].

We observed more variation in adherence to guidelines for less common cancers, such as urologic or pancreatic, where screening recommendations are more complex and often require additional factors such as family cancer history in addition to gene variant status [11, 12]. This complexity can lead to inconsistent application of guidelines, resulting in both under- and over-utilization of screenings.

Over-utilization, where screenings exceed recommended frequencies, was prevalent in our cohort. For example, we found that 19% of patients underwent more frequent screenings than necessary. The harm caused by over-utilization is multifaceted: it can lead to physical harm through unnecessary procedures, psychological harm due to stress and anxiety, and financial harm from increased medical bills [13].

Under-utilization of care, where patients do not receive recommended screenings, was also prevalent in our cohort, with 25% of patients receiving less frequent screenings than recommended. The consequences of under-utilization are severe, as delayed or missed cancer diagnoses can result in worse prognosis and reduced survival rates.

Our findings should be interpreted in the context of this specific U.S. integrated healthcare system. International guidelines, including those from the European Hereditary Tumour Group (EHTG), offer different recommendations based on accumulated evidence from the Prospective Lynch Syndrome Database (PLSD) [14]. PLSD data demonstrate that cancer risks, optimal surveillance intervals, and mortality benefits vary substantially by specific gene variant, and that guidelines should be interpreted with awareness of these gene-specific differences [15].

Hereditary Breast and Ovarian Cancer (HBOC) syndrome and Lynch syndrome are both recognized by the CDC as Tier 1 genomic conditions with significant potential for positive impact on population health management when detected early and intervened upon [16]. Similar system-level barriers affecting surveillance adherence have been documented across hereditary cancer syndromes, including insurance coverage gaps, provider knowledge deficits, and lack of coordinated care systems [17].

Our study has several limitations. The sample size was small for specific variant types. The study was unable to control for site- and provider-specific counseling practices, which could introduce variability in how patients were informed about and engaged in their screening protocols. We were unable to assess specific reasons for non-concordance (patient choice vs. provider recommendation vs. access barriers). Our findings from a single integrated U.S. healthcare system may not generalize to other healthcare settings or populations. The high proportion of PMS2 carriers (35%) in our cohort, which is higher than typically reported in Lynch syndrome cohorts, likely reflects ascertainment through universal tumor testing and may limit comparability to studies using different identification methods.

A strength of our study is its applicability to real-world practice in an integrated healthcare system. The data collection was based on review of medical records rather than patient recall or coding, ensuring a higher level of accuracy and reliability. The study assessed multiple modalities of surveillance across various cancer types, offering a comprehensive perspective on utilization of screening practices.

Lynch Syndrome is a complex hereditary cancer syndrome with constant evolution of screening recommendations as research is published. This is one of the few studies to evaluate longitudinally the rate of screening performed in this population. Given the high rate of primary cancers diagnosed prior to genetic testing, there is an opportunity for improving identification of Lynch syndrome patients unaffected by cancer. Given low levels of concordance with recommended guidelines, following strategies used for HBOC, such as referrals to hereditary cancer centers of excellence with coordinated care systems and reminder mechanisms, may improve screening adherence.

## Conclusions

Only 48% of patients in this integrated healthcare system received guideline-concordant Lynch syndrome surveillance during the study period. Our findings highlight variation in screening practices and adherence to NCCN guidelines among patients with different pathogenic variants. Barriers to concordant care likely exist at patient, provider, and healthcare system levels. Future efforts should focus on developing coordinated management systems, improving provider education about variant-specific recommendations, and addressing system-level barriers to appropriate surveillance.

## Data Availability

The datasets used and/or analyzed during the current study are available from the corresponding author on reasonable request.

## References

[1] Auranen A, Joutsiniemi T. A systematic review of gynecological cancer surveillance in women belonging to hereditary nonpolyposis colorectal cancer (Lynch syndrome) families. Acta Obstet Gynecol Scand. 2011. 10.1111/j.1600-0412.2011.01091. 21306348 10.1111/j.1600-0412.2011.01091.x

[2] Win AK, et al. Prevalence and penetrance of major genes and polygenes for colorectal cancer. Cancer Epidemiol Biomarkers Prev. 2017. 10.1158/1055-9965.EPI-16-0693. 27799157 10.1158/1055-9965.EPI-16-0693 PMC5336409

[3] Bonadona V, et al. Cancer risks associated with germline mutations in MLH1, MSH2, and MSH6 genes in Lynch syndrome. JAMA. 2011. 10.1001/jama.2011.743. 21642682 10.1001/jama.2011.743

[4] Chen LM, et al. Gynecologic cancer prevention in Lynch syndrome/hereditary nonpolyposis colorectal cancer families. Obstet Gynecol. 2007. 10.1097/01.AOG.0000267500.27329.85. 17601891 10.1097/01.AOG.0000267500.27329.85

[5] Gupta S, Weiss JM, et al. Genetic/familial high-risk assessment: colorectal. National Comprehensive Cancer Network (NCCN); 2023. https://www.nccn.org/professionals/physician_gls/pdf/genetics_colon.pdf

[6] Provenzale D, et al. Genetic/familial high-risk assessment: colorectal version 1.2016, NCCN clinical practice guidelines in oncology. J Natl Compr Canc Netw. 2016;14(8):1010–30. 10.6004/jnccn.2016.0108. 27496117 10.6004/jnccn.2016.0108

[7] Yurgelun MB, Hampel H. Recent advances in Lynch syndrome: diagnosis, treatment, and cancer prevention. Am Soc Clin Oncol Educ Book. 2018. 10.1200/EDBK_208341. 10.1200/EDBK_208341 30231390

[8] Dominguez-Valentin M, et al. Risk-reducing hysterectomy and bilateral salpingo-oophorectomy in female heterozygotes of pathogenic mismatch repair variants: a prospective Lynch syndrome database report. Genet Med. 2021;23(4):705–12. 10.1038/s41436-020-01029-1. 33257847 10.1038/s41436-020-01029-1 PMC8026395

[9] Kaiser Permanente. Fast facts; 2024. https://about.kaiserpermanente.org/who-we-are/fast-facts

[10] Watkins KE, et al. Colorectal cancer screening in a large healthcare system: surveillance adherence, outcomes, and missed cases. Hered Cancer Clin Pract. 2011;9:8. 10.1186/1897-4287-9-8. 21899746 10.1186/1897-4287-9-8 PMC3180430

[11] Mork M, et al. Lynch syndrome: a primer for urologists and panel recommendations. J Urol. 2015. 10.1016/j.juro.2015.02.081. 25711197 10.1016/j.juro.2015.02.081

[12] Kastrinos F, et al. Risk of pancreatic cancer in families with Lynch syndrome. JAMA. 2009. 10.1001/jama.2009.1529. 19861671 10.1001/jama.2009.1529 PMC4091624

[13] Korenstein D, et al. Development of a conceptual map of negative consequences for patients of overuse of medical tests and treatments. JAMA Intern Med. 2018. 10.1001/jamainternmed.2018.3573. 30105371 10.1001/jamainternmed.2018.3573 PMC7505335

[14] Møller P, et al. Dominantly inherited micro-satellite instable cancer – the four Lynch syndromes. Hered Cancer Clin Pract. 2023;21:19. 10.1186/s13053-023-00263-3. 37821984 10.1186/s13053-023-00263-3 PMC10568908

[15] Dominguez-Valentin M, et al. Mortality by age, gene and gender in carriers of pathogenic mismatch repair gene variants receiving surveillance for early cancer diagnosis and treatment: a report from the Prospective Lynch Syndrome Database. eClinicalMedicine. 2023;58:101909. 10.1016/j.eclinm.2023.101909. 37181409 10.1016/j.eclinm.2023.101909 PMC10166779

[16] Centers for Disease Control. Tier 1 genomics applications and their importance to public health. https://archive.cdc.gov/www_cdc_gov/genomics/implementation/toolkit/tier1.htm

[17] Bellcross C, et al. Implementing screening for Lynch syndrome among patients with newly diagnosed colorectal cancer. Genet Med. 2012;14:152–62. 10.1038/gim.0b013e31823375ea. 22237445 10.1038/gim.0b013e31823375ea PMC3762677

